# Transcripts enriched in codons that trigger P‐site tRNA‐mediated mRNA decay possess stable mRNA

**DOI:** 10.1002/2211-5463.70277

**Published:** 2026-06-12

**Authors:** Rodolfo Lopes Carneiro, Fernando Lucas Palhano

**Affiliations:** ^1^ Programa de Biologia Estrutural, Instituto de Bioquímica Médica Leopoldo de Meis Universidade Federal do Rio de Janeiro Brazil

**Keywords:** codon usage, mRNA decay, mRNA turnover, Translation, tRNA

## Abstract

Synonymous codon usage significantly influences mRNA stability in yeast by guiding mRNA decay during translation. The CCR4‐NOT complex is central to this process, interacting with ribosomes when the A and E sites are unoccupied, a state that arises when a nonoptimal codon with low tRNA availability is at the A site. This triggers recruitment of decay factors, reducing the stability of transcripts enriched in such codons. In humans, codon‐mediated mRNA decay is less well‐understood. Recent research has identified a related but distinct mechanism called P‐site tRNA‐mediated decay (PTMD). Unlike yeast, human CCR4‐NOT recruitment depends on specific arginine codons (CGG, CGA, or AGG) at the P site and slow decoding at the A site, allowing E‐site vacancy and CNOT3‐dependent binding. Through analysis of public datasets, we explored the characteristics of human transcripts enriched in PTMD codons. Interestingly, these codons are mostly found in transcripts with longer half‐lives. This suggests that, rather than targeting already unstable mRNAs as in yeast, PTMD in humans selectively reduces the stability of otherwise long‐lived transcripts, indicating a regulatory role distinct from the decay associated with codon usage.

## Graphical abstract

PTMD codons were first described by Mendel *et al.* as mediators of an mRNA decay pathway dependent on the human protein CNOT3, homologous to yeast Not5. Our findings confirm that PTMD codons destabilize transcripts; however, unlike in yeast, the human pathway specifically targets and slightly destabilizes primarily stable mRNAs.

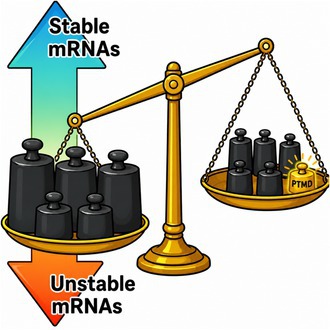

**Abbreviations**
CAI: Codon Adaptation Index
CDS: Coding Sequence
CNOT3‐IP: CNOT3 immunoprecipitation
CNOT3‐KO: CNOT3 knockout
DT: dwell time
mRNA: messenger RNA
ORF: Open Reading Frame
PTMD: P‐site tRNA‐mediated decay
tRNA: transfer RNA

Precise control of messenger RNA (mRNA) turnover is a key regulatory mechanism in numerous cellular processes. Some of these are inherent to mRNA function—for instance, transcripts with longer half‐lives tend to accumulate in the cytoplasm and are translated into highly expressed proteins. Additionally, mRNA decay regulation has been linked to the immune response [1], responses to mRNA damage [2, 3, 4, 5], and cell cycle progression and differentiation [6, 7, 8]. Various molecular machineries regulate mRNA decay, acting either globally or targeting specific motifs in transcript sequences [9].

The canonical pathway of mRNA degradation begins with deadenylation at the 3′ end by the Pan2–Pan3 and CCR4–NOT complexes [10, 11], followed by decapping at the 5′ end by the Dcp1–Dcp2 complex. Once the mRNA ends are exposed, the exonuclease Xrn1 degrades the transcript in the 5′ to 3′ direction [12], while the Ski complex facilitates 3′ to 5′ degradation [13, 14].

Although this canonical pathway is largely translation‐independent, increasing attention has been given to translation‐dependent regulation of mRNA stability, especially through synonymous codon usage. Despite the degeneracy of the genetic code, synonymous codons can influence mRNA half‐life and protein expression in yeast [15]. Between the terms used to refer to codon usage, one key term is ‘codon optimality’, first defined in 2009 by Zhou, Weems and Wilke [16], as ‘the odds ratio of codon usage between highly and lowly expressed groups [of genes]’. Nevertheless, ‘optimality’ has been used since at least 1985 to denote how well a codon‐anticodon interaction occurs during translation, considering mainly transfer RNA (tRNA) availability [17]. Some codons are considered optimal because they match more abundant tRNAs, which leads to faster and more accurate translation. Others are nonoptimal, being decoded by less abundant tRNAs, leading to a translation that is slower and more prone to errors [17]. Presnyak *et al*. demonstrated that synonymous codon optimality is a major determinant of mRNA stability. This relationship is mediated by the CCR4‐NOT complex [16, 17, 18, 19]. In yeast, the Not5 subunit of the CCR4‐NOT complex interacts with the ribosomal E‐site when both the A and E sites are unoccupied—a ribosome conformation that arises during slow decoding of nonoptimal codons. This interaction promotes recruitment of the decapping factor Dhh1 and subsequent mRNA decay [19, 20].

While the relation between codon optimality and mRNA decay is best characterized in yeast, it appears to be conserved across multiple species, including *Escherichia coli* [21], zebrafish [7, 8], *Trypanosoma brucei* [22, 23], *Drosophila melanogaster* [24], and humans [25, 26].

In humans, however, the molecular mechanisms differ in key ways. A recent study revealed that CNOT3, the human homolog of yeast Not5, targets transcripts based on the structure of the tRNA occupying the ribosomal P‐site [27]. Using ribosome footprint profiling in HEK293T cells, the authors showed that CNOT3 preferentially associates with ribosomes translating three specific arginine codons—CGA, CGG, and AGG—when both the A and E sites are vacant. This process, termed P‐site tRNA‐mediated decay (PTMD), requires both the presence of one of these codons in the P‐site and slow decoding at the A‐site to enable E‐site vacancy and CCR4‐NOT recruitment. Knockout of CNOT3 stabilizes numerous transcripts enriched in these codons, supporting its role in mRNA destabilization. PTMD is a codon‐specific mRNA decay pathway [27]. The molecular mechanism that explains why CGA, CGG, and AGG codons trigger CCR4‐NOT recruitment relies on the interactions between CNOT3, a member of the CCR4‐NOT complex, and PTMD codons. Hydrogen bonding interactions with the D‐arm of PTMD in the P‐site promote CNOT3 recruitment and mRNA degradation [27].

Despite these findings, it remains unclear whether transcripts enriched in PTMD codons differ in codon composition and mRNA stability compared to other transcripts. Moreover, the extent to which PTMD codons followed by poorly decoded codons influences transcript half‐life is still not understood.

In this study, we investigate the genomic landscape of PTMD regulation by analyzing genes enriched in CGA, CGG, and AGG codons. Using datasets from Zhu *et al*. [27] and additional sources, we show that PTMD‐target codons are primarily found in transcripts with long half‐lives, whereas unstable transcripts tend to lack PTMD codons. We also observed that potentially PTMD‐target transcripts (rich in PTMD codons) have no preference for codons with low codon dwell time (fast translating) or high dwell time (slow translation) on the A‐site followed by the PTMD codon. Moreover, the presence of low or high codon dwell time on the A‐site followed by the PTMD codon has no effect on transcript stability. These findings suggest that PTMD has a mild impact in reducing half‐lives of stable transcripts, rather than mediating decay of inherently unstable mRNAs.

## Methods

### Data sources

Coding sequences (CDS) and gene annotations for *Homo sapiens* GRCh38 were downloaded from Ensembl. Transcripts were filtered to include only those with lengths divisible by three and ending with a canonical stop codon. To avoid redundancy and overrepresentation of genes with multiple isoforms, only the longest transcript of each gene was retained. Of the 23 151 genes in the dataset, 17 324 (74.8%) had multiple annotated transcripts, with an average of 5.5 transcripts per gene. The selected longest transcripts had an average length of 564 codons and an average PTMD score of 0.193. In contrast, the discarded transcripts had an average length of 409 codons and an average PTMD score of 0.196.

**Fig. 1:**
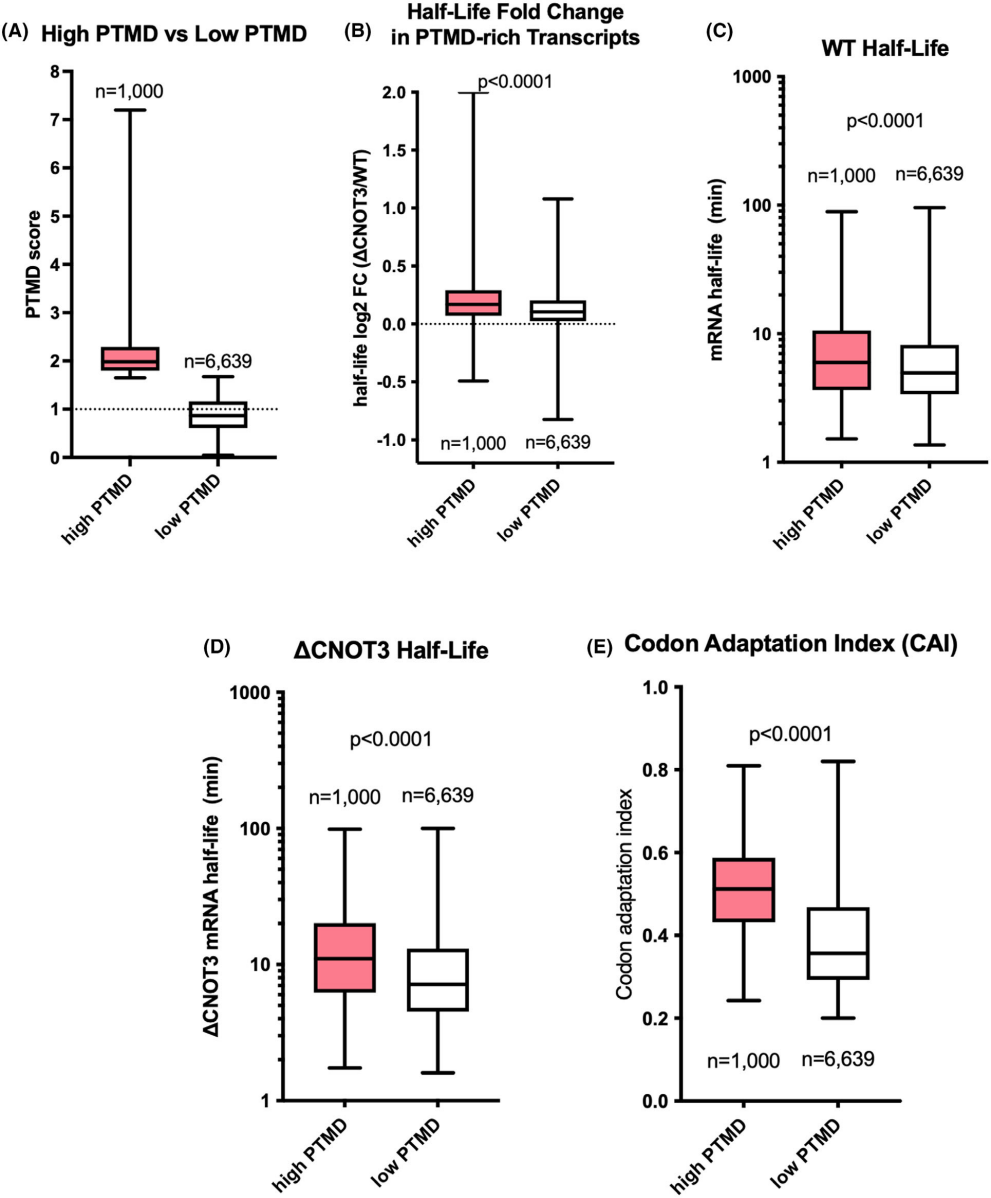
Comparison of PTMD score and transcripts rich in AGG, CGA, and CGG with other metrics. (A) Transcripts with high PTMD scores and other transcripts were split by their PTMD scores. (B) Log2 of the mRNA half‐life fold change between CNOT3‐knockout cells and wild‐type cells. (C) mRNA half‐life of transcripts with high PTMD score compared to mRNA half‐life of the remaining transcripts in wild‐type cells. (D) mRNA half‐life of transcripts with high PTMD score compared to mRNA half‐life of the remaining transcripts in CNOT3‐knockout cells. (E) Codon Adaptation Index in transcripts with high PTMD score vs transcripts with low PTMD score. The top whisker, top of the box, line inside the box, bottom of the box, and bottom whisker represent the maximum value, the upper quartile (75th percentile), the median, the lower quartile (25th percentile), and the minimum value, respectively. *P*‐values were given by the Kolmogorov–Smirnov test.

Dwell time values correspond to the translation elongation times assigned to each of the 61 sense codons, as reported by Narula *et al*. [28], while the tRNA abundance values used here were obtained from Behrens *et al*. [29] (Figs 4 and 5). Alternatively, dwell time and tRNA abundance were obtained from [30] (Figs S3 and S4). The Codon Adaptation Index (CAI) values used in Fig. 1E were obtained from Sharp & Li [31]. Ribosome profiling data for calculating gene enrichment under CNOT3‐IP was obtained from Zhu *et al*. [27].

**Fig. 2:**
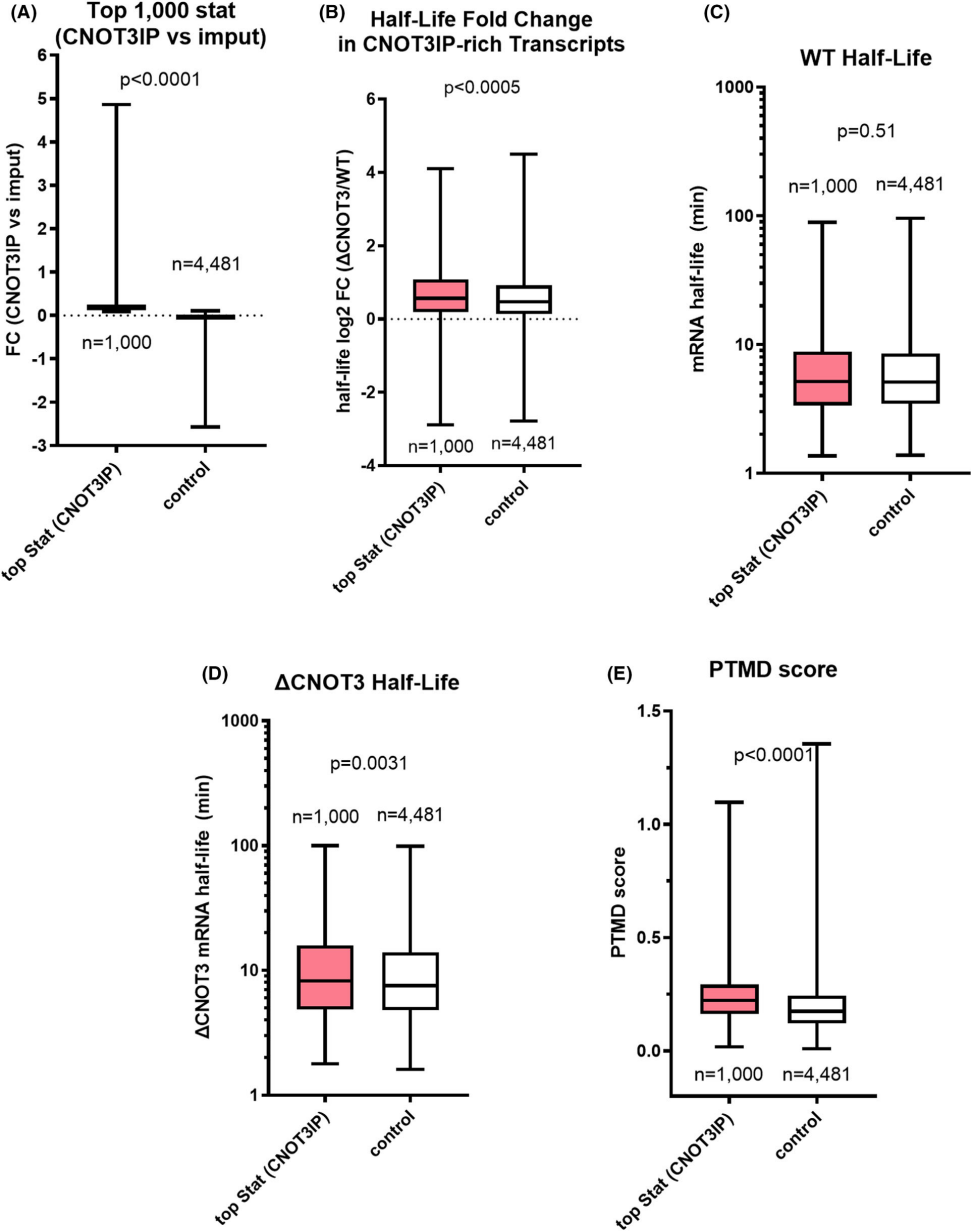
Comparison of enrichment under CNOT3‐IP and mRNA half‐life. (A) Transcripts with high stat (fold change divided by the standard error) and other transcripts were split by their CNOT3‐IP score. (B) mRNA half‐life Fold Change between CNOT3‐KO cells and wild‐type cells, for two groups of genes, one of genes enriched under CNOT3‐IP (n = 1000), and the other with genes not enriched under CNOT3‐IP (n = 4481). Kolmogorov–Smirnov's *P* < 0.0001. (C) mRNA half‐life of wild‐type cells, for two groups of genes, one of genes enriched under CNOT3‐IP and the other with genes not enriched under CNOT3‐IP. Kolmogorov–Smirnov's *P* = 0.51. (D) mRNA half‐life of CNOT3‐KO cells, for two groups of genes, one of genes enriched under CNOT3‐IP and the other with genes not enriched under CNOT3‐IP. Kolmogorov–Smirnov's *P* = 0.0031. (E) PTMD score in transcripts with high CNOT3‐IP score vs transcripts with low CNOT3‐IP score. The top whisker, top of the box, line inside the box, bottom of the box, and bottom whisker represent the maximum value, the upper quartile (75th percentile), the median, the lower quartile (25th percentile), and the minimum value, respectively.

**Fig. 3:**
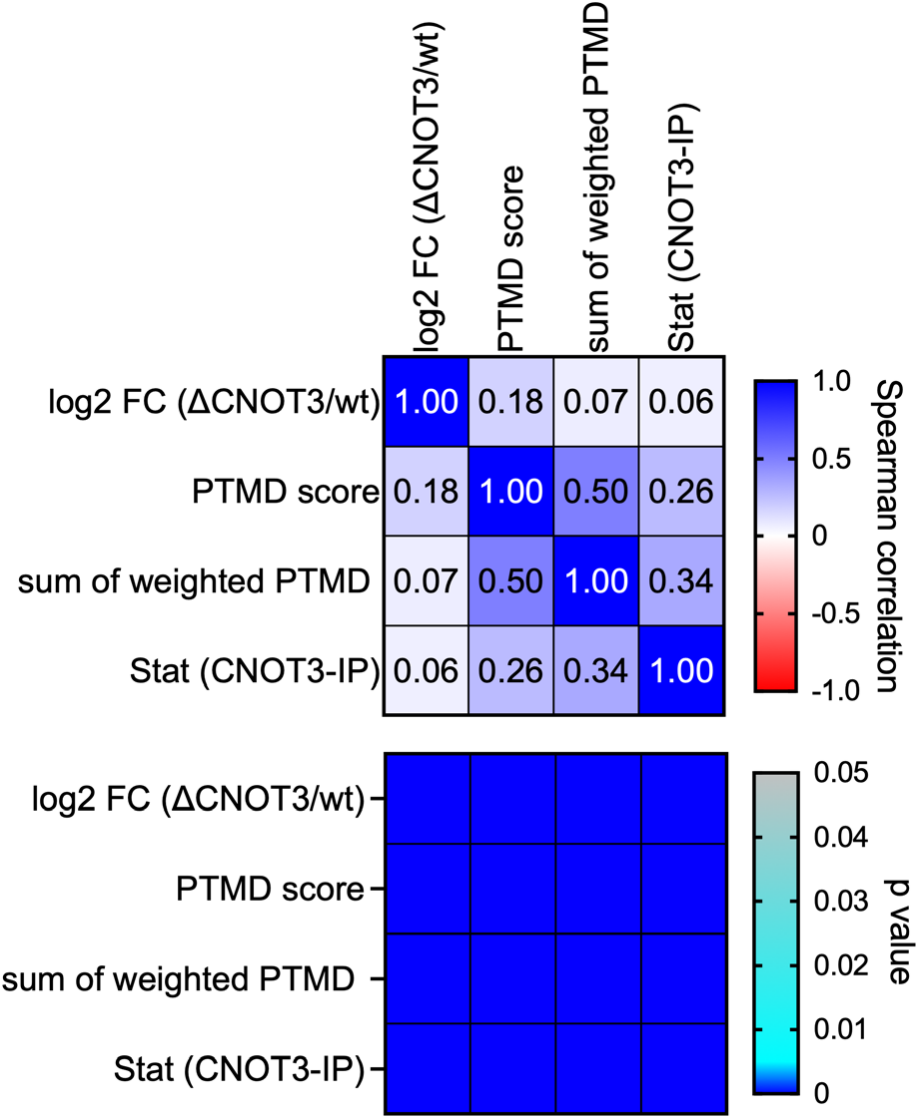
Spearman correlation matrix of four parameters: mRNA half‐life after CNOT3 knockout relative to the wild‐type cells (log 2 FC (CNOT3/wt)), PTMD score, PTMD score without a normalization by the length of the ORF (sum of weighted PTMD), and CNOT3‐IP enrichment (Stat CNOT3‐IP).

### CNOT3‐IP metrics and group stratification

Ribo‐seq samples (two inputs and two under CNOT3‐IP) were aligned to the annotated exons of the human genome using STAR. If a gene had less than 10 reads aligned to it in any sample, the gene was discarded. Each read was attributed only to a single gene. Then, we used DeSeq2 to analyze the enrichment between CNOT3‐IP samples, compared to the input samples. From there, we used the adjusted *P*‐value and stat metrics for further analysis. Specifically, the adjusted *P*‐value was used to infer which genes were significantly enriched. The stat metric, given by the ratio between the Fold Change and the standard error, was used for stratifying the groups used in further analysis.

### Codon pair occurrence calculations

To quantify codon pair occurrence, we developed a script that enumerates the absolute frequency of each codon pair across the human transcriptome, considering all 3904 possible combinations (61 × 64, excluding stop codons in the first position). Codon pairs were counted in‐frame at any position within each transcript's open reading frame (ORF).

**Fig. 4:**
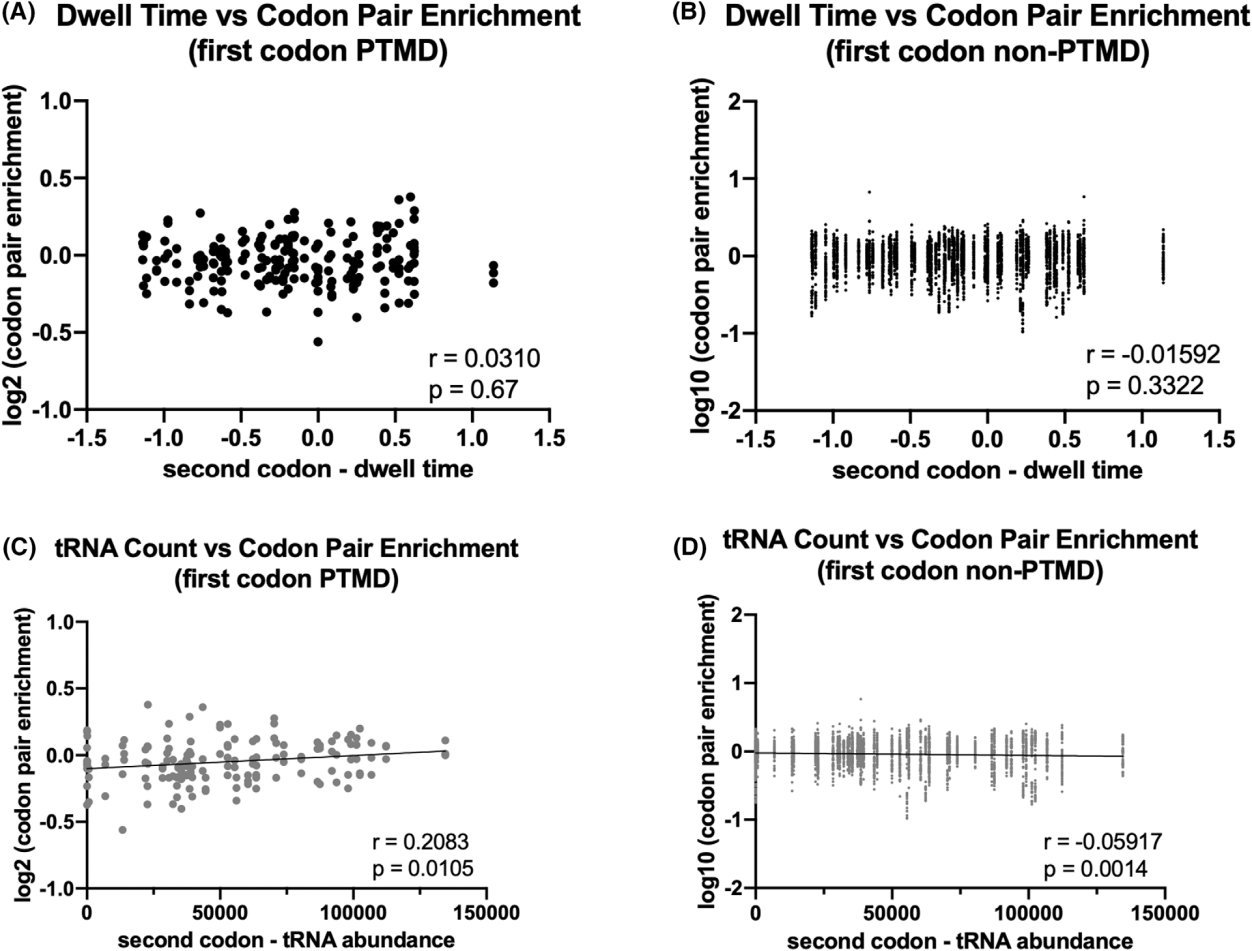
Correlations of PTMD occurrence, ribosome dwell time, and tRNA. (A) Correlation between codon pair occurrence in the genome ORFs, when the first codon of the pair is a PTMD codon, and the dwell time of the second codon in the pair. (B) Correlation between codon pair occurrence in the genome ORFs, when the first codon of the pair is a codon other than a PTMD one, and the dwell time of the second codon in the pair. (C) Correlation between codon pair enrichment in the genome ORFs, when the first codon of the pair is a PTMD codon, and the tRNA count for the anticodon correspondent to the second codon in the pair. (D) Correlation between codon pair occurrence in the genome ORFs, when the first codon of the pair is a codon other than a PTMD one, and the tRNA abundance for the anticodon correspondent to the second codon in the pair. Dwell time and tRNA abundance were obtained from [29, 32] respectively. *P*‐values were given by Pearson's correlation index (r).

The occurrence values used in Fig. 4 were obtained by dividing this absolute frequency by the pair expected frequency, given by the product of each codon frequency, as follows:

$${\text{occurrence}}_{\mathrm{ij}}=\frac{{\text{Npair}}_{\mathrm{ij}}}{N_{i}\cdot N_{j}}$$

where *Npair*_*ij*_ is the frequency of the codon pair composed of codons *i* and *j*, and *N*_*i*_ and *N*_*j*_ represent the individual frequencies of codons *i* and *j*.

The data used for Fig. 4 are available in Table S1.

### PTMD score

Zhu *et al*. [27] measured, through ribosome profiling, how often each codon was found occupying the ribosome P‐site. This was done under CNOT3 immunoprecipitation and also in standard ribo‐seq, and these values were used to calculate enrichment values for each codon. These values were called, here, as ‘weights’ for the calculation of the PTMD score.

The PTMD score of a transcript was defined as the occurrence of AGG, CGA, and CGG multiplied by the weight of the respective codon and divided by the transcript length in codons, following the equation:

$${\text{score}}_{i}=\frac{\sum_{j}\left(N_{\mathrm{ij}}\cdot W_{j}\right)}{L_{i}}$$

where *score*_*i*_ is the PTMD score of the transcript *i*, *N*_*ij*_ is the number of times the codon *j* is found in the transcript *i*, *W*_*j*_ is the weight of the codon *j* as described above, and *L* is the length of transcript *i*, in codons.

### Search for PTMD codons and A‐site codons following the PTMD codons

**Fig. 5:**
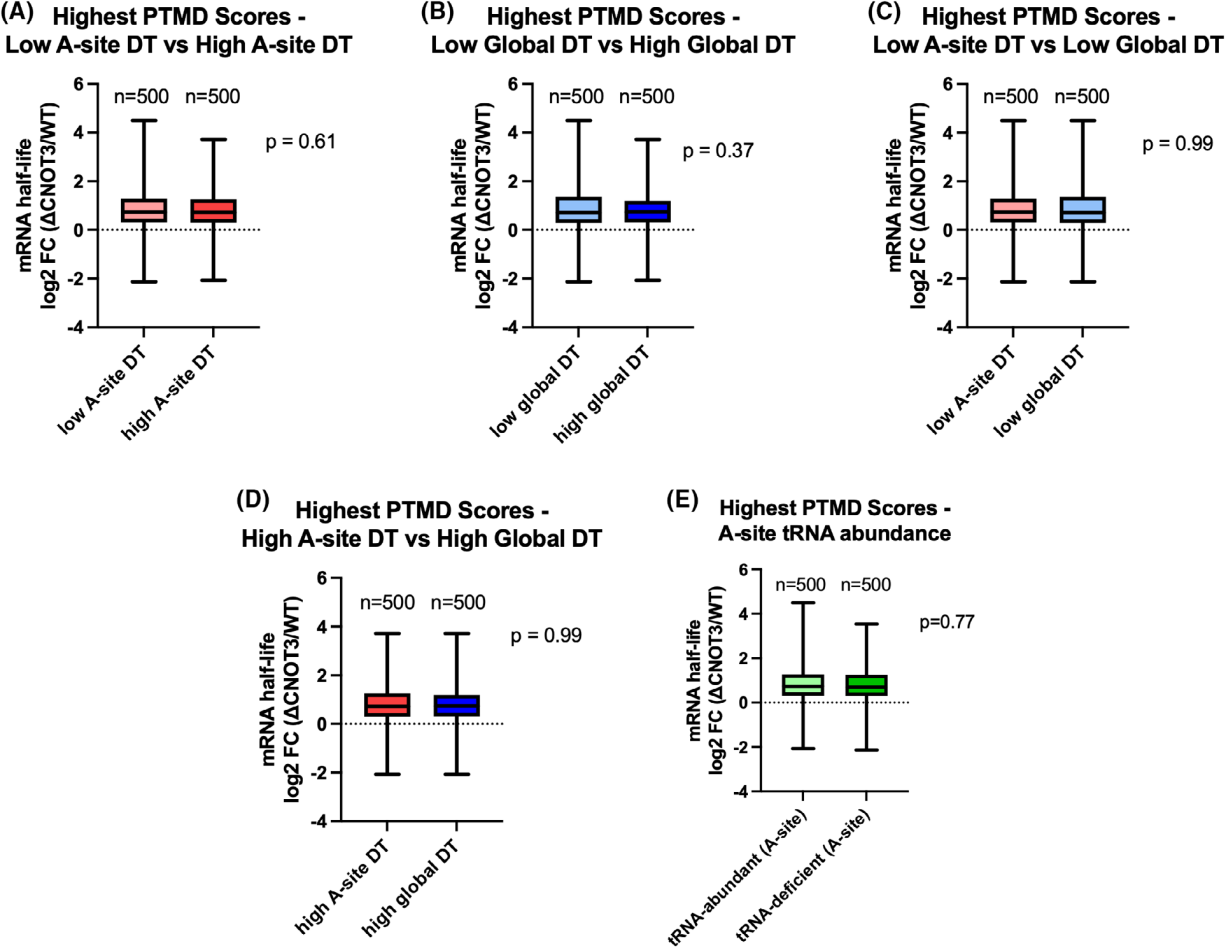
Dwell Time and tRNA availability of codons on the A‐site have no impact on P‐site tRNA‐mediated decay. (A) mRNA half‐life fold change (ΔCNOT3/WT) of transcripts with high PTMD score comparing the lowest dwell time in the A‐site after CGA, CGG, or AGG vs transcripts with the highest dwell time in the A‐site after CGA, CGG, or AGG. (B) mRNA half‐life fold change (ΔCNOT3/WT) of transcripts with high PTMD score, comparing the median dwell time for all codons throughout the transcript, instead of only the codons on the A‐site following PTMD codons. (C) mRNA half‐life fold change (ΔCNOT3/WT) of transcripts with high PTMD score, comparing transcripts with low dwell time on the A‐sites following PTMD codons to the transcripts with low median dwell time for all the codons throughout the transcript. (D) mRNA half‐life fold change (ΔCNOT3/WT) of transcripts with high PTMD score, comparing transcripts with high dwell time on the A‐sites following PTMD codons to the transcripts with high median dwell time for all the codons throughout the transcript. (E) Same analysis as (A) but using tRNA abundance in place of dwell time. *P*‐values given by Kolmogorov–Smirnov test. Dwell time and tRNA abundance were obtained from [29, 32] respectively. The top whisker, top of the box, line inside the box, bottom of the box, and bottom whisker represent the maximum value, the upper quartile (75th percentile), the median, the lower quartile (25th percentile), and the minimum value, respectively.

To identify transcripts containing PTMD target codons (AGG, CGA, or CGG) and the codons that appear immediately after them, we implemented a Python script that scans each coding sequence in our human transcriptome library. For every in‐frame codon, the subsequent codon was considered to occupy the A‐site relative to that P‐site codon. In Fig. 5, the dwell time and tRNA abundance values represent the median values of codons found in the A‐site following each occurrence of AGG, CGA, or CGG. The median was chosen as the primary measure of central tendency due to the typically low frequency of PTMD codons per transcript, which can make the mean sensitive to outliers.

The data used for Fig. 1A–D and Fig. 3 is available in Table S2.

## Results

### PTMD codons are enriched in stable transcripts

To investigate the possible roles of PTMD codons in the translation dynamics, particularly their effect on mRNA stability, we first quantified the frequency of the CGG, CGA, and AGG (PTMD codons) within each transcript. Following the methodology used by Mendell's group, we calculated a PTMD score for each transcript, weighting the CGG, CGA, and AGG codons by their enrichment in the P‐site of CNOT3‐associated ribosomes and normalizing by the total number of codons in the ORF [27]. This measure was defined as the transcript's PTMD score. We then stratified the transcripts into two groups: the 1000 transcripts with the highest PTMD scores (high PTMD group) and all remaining transcripts (low PTMD group) (Fig. 1A). We then checked if transcripts with high PTMD scores would have more stable mRNAs after CNOT3 knockout, if compared to genes with lower PTMD scores, using half‐life data from Zhu *et al*. [27]. The high PTMD group presented a higher increase in mRNA half‐life after CNOT3 knockout (CNOT3‐KO) relative to the wild‐type cells (Fig. 1B), corroborating what was previously described [27]. Mendell's group showed that the several mRNAs being destabilized by PTMD are mRNAs for highly expressed mitochondrial proteins [27]. To address whether the translation pauses caused by signal peptides in transcripts addressing the mitochondrion would explain the result observed in Fig. 1B, we performed the same analysis now discarding mitochondrial proteins. The difference between the two groups remains significant suggesting that PTMD codons, and not the signal peptides translation, are responsible for CNOT3 recruitment (Fig. S1). Interestingly, the high PTMD group also presented more stable mRNAs compared to the low‐PTMD group, in both wild‐type cells (Fig. 1C) and CNOT3‐KO cells (Fig. 1D).

Our data indicate that PTMD codons are more prevalent in stable mRNAs, instead of unstable ones, despite having a destabilizing effect. One possible explanation for this stability is the possibility of these transcripts being enriched in codons with a stabilizing effect. Codon usage is known to influence mRNA stability, as codons that are more efficiently decoded tend to correlate positively with longer mRNA half‐lives [26]. This property is often referred to as codon optimality. Several approaches can be used to estimate codon optimality depending on the context [16, 26, 32]. In this study, we used the codon adaptation index (CAI) as a proxy for codon optimality [32]. The CAI reflects how closely a transcript's codon usage matches that of highly expressed human genes, thereby serving as an indicator of its potential translational efficiency [33]. Interestingly, transcripts in the high PTMD group also displayed higher CAI values than those in the low PTMD group (Fig. 1E).

Next, we checked whether the genes enriched in PTMD codons would be the same that recruited CNOT3 under translation. The first step was to check whether our data replicated the codon enrichment in CGA, CGG, and AGG at the P‐site, as described by Zhu *et al*. [27]. After processing their ribosome profiling raw data, we achieved very proximate results (Fig. S2). Then, we assessed the differential expression of genes between the two ribosome profiling samples—with and without CNOT3 immunoprecipitation (CNOT3‐IP). Genes that presented a number of reads too low (< 10 reads) to perform a differential expression analysis were excluded from the analysis, resulting in a sample of 5481 genes for which we also had mRNA half‐life data. When assessing gene enrichment, contrary to our expectations, only 17 genes were enriched under CNOT3‐IP (Fig. S3). This probably was due to the low coverage of the CNOT3‐IP ribosome profiling samples. Interestingly, the fourth most enriched gene under CNOT3‐IP was TNKS1BP1 (also known as TAB182), whose protein has been reported to be associated with the CCR4‐NOT complex [34, 35]. Since our data relate to the RNA molecules being translated by ribosomes coimmunoprecipitated with CNOT3, the most reasonable explanations for this finding are that the CCR4‐NOT complex regulates its own translation as a feedback response, or that the TNKS1BP1 protein recruits the complex during its own translation.

Since working with a group of only 17 genes in a universe of 5481 genes is unfeasible, we chose the top 1000 mRNAs that were enriched in the CNOT3 immunoprecipitation. Specifically, we selected the 1000 genes with the highest stat variable (fold change divided by the standard error) in the enrichment analysis. When comparing this set to the genes with the top 1000 genes with highest PTMD scores, only 279 genes were shared between the two groups. This overlap is a small fraction of each group, but it is 1.53 times greater than expected by chance (182 genes), which is statistically significant (*P*‐value < 0.001 on a Monte Carlo simulation).

Then, we compared the top 1000 CNOT3‐IP group of genes with all the genes (4481) considered as not enriched under CNOT3‐IP (Fig. 2A). We observed that the increment of the half‐lives of CNOT3‐KO cells, relative to the wild‐type cells, was bigger in the CNOT3‐IP group if compared to the group of genes not enriched under CNOT3‐IP (Fig. 2B). Nonetheless, there was no difference between the two groups when accounting for the half‐lives of the wild‐type cells alone (Fig. 2C). The observed change in mRNA half‐lives in Fig. 2B could be credited exclusively to the increase of their mRNA half‐lives after CNOT3 deletion (Fig. 2D). The CNOT3‐IP enrichment genes presented an increased PTMD score when compared to the control (Fig. 2E).

The relationship among CNOT3‐IP enrichment, PTMD score and mRNA half‐life after CNOT3 knockout relative to the wild‐type cells was analyzed by a correlation matrix (Fig. 3). The correlation of mRNAs stability after CNOT3 knockout with PTMD score was 0.18 while with CNOT3‐IP enrichment was just 0.06 (Fig. 3). To focus more on absolute values rather than normalized values, we also included in this analysis another metric, which we called the weighted PTMD sum. This metric is equivalent to the PTMD score, but without normalization by ORF length. Nevertheless, the sum of weighted PTMD correlates poorly with mRNAs stability after CNOT3 knockout (Fig. 3). Together, this experiment revealed that the parameter analyzed herein that best correlates with mRNA stability after CNOT3 knockout was the PTMD score and not CNOT3‐IP enrichment. One possible explanation is the low coverage of the ribosome profiling data under CNOT3‐IP, which was used to calculate enrichment. Therefore, we focused our subsequent analyses on the PTMD score metric, as it showed a stronger correlation with mRNA stability than the enrichment under CNOT3‐IP.

In summary, we observed that not every gene enriched in PTMD codons is associated with CNOT3 recruitment. Moreover, we observed that transcripts enriched in PTMD codons are more stable and exhibit higher CAI values compared to the genome. Some of these transcripts show greater mRNA stabilization upon CNOT3 deletion, suggesting that they are preferential targets of the CCR4‐NOT complex.

### Analyzing the dwell time and tRNA abundance of codons on the A‐site following PTMD codons on the P‐site

One of the key aspects of the PTMD mechanism is that CNOT3 binds to the ribosome only when both the E‐site and A‐site are vacant. Zhu *et al*. proposed that this occurs when the A‐site codon has a long dwell time, giving enough time for the tRNA in the E‐site to dissociate before a new aminoacyl‐tRNA arrives at the A‐site.

To explore whether this feature might have been evolutionarily selected as a mechanism for mRNA decay control, we investigated whether the dwell time of each codon correlates with how frequently it appears after the codons AGG, CGA, or CGG in the genome.

To do this, we developed a script to calculate the observed frequency of each codon pair in the genome and compare it to the expected frequency based on the individual usage of each codon. We refer to this metric as codon pair enrichment (shown on the y‐axis of all panels in Fig. 4).

In Fig. 4A, codon pair enrichment was calculated for all 183 combinations (3 × 61) of codons, with one PTMD codon (AGG, CGA, or CGG) fixed in the first position. The x‐axis represents the dwell time of the codon that follows the PTMD codon, as measured by ribosome profiling of HEK293T cells [28].

We found no correlation between the second codon's dwell time and codon pair enrichment, regardless of whether the first codon was AGG, CGA, or CGG (Fig. 4A) or any other codon (Fig. 4B).

In contrast, when we used tRNA abundance [29] instead of dwell time, a statistically significant positive correlation (Pearson's r > 0.2) was observed for codons following AGG, CGA, or CGG (Fig. 4C). For codons following non‐PTMD codons, a weaker but significant negative correlation was found (Fig. 4D).

Finally, these effects appear to be specific to codons that follow AGG, CGA, or CGG and not due to general proximity. When these PTMD codons were placed in the second position instead of the first, the correlations disappeared (Fig. S4).

A similar trend was observed when we used dwell time and tRNA abundance data from another study (Fig. S5) that analyzed hiPSC‐derived neuronal cells [30]. We conclude that PTMD codons tend to be followed by codons with high tRNA abundance (Fig. 4C), although no bias was detected regarding the decoding time of these codons (Fig. 4A).

### CNOT3‐associated mRNA decay is not dependent on ribosomal dwell time

To further clarify the impact of dwell time as well tRNA abundance on mRNA decay through PTMD, another script was used to search for each AGG, CGA, and CGG in each transcript and calculate the average dwell time and average tRNA abundance of the codons following them. Then, we selected the 1000 transcripts with the highest PTMD scores, the same ones as Fig. 1A, but we stratified them into two groups based on the median dwell time of the codons following AGG, CGA, and CGG. For simplicity, these groups were called ‘high A‐site DT’ and ‘low A‐site DT’, since the PTMD machinery is supposed to act when these codons with extreme dwell time values occupy the A‐site of the ribosome. After stratifying our sample, we compared the mRNA half‐life increase under CNOT3 knockout, in comparison with the wild‐type, between those two groups. Unexpectedly, the two groups showed no statistically significant difference (Fig. 5A).

We next repeated the analysis, but stratifying the transcripts by the dwell time of every codon in their transcripts, instead of only the codons following AGG, CGA, and CGG. Again, the two groups showed no difference in the increase of mRNA half‐life under knockout of CNOT3 (Fig. 5B). Subsequent comparisons between the groups with slow dwell time (Fig. 5C) or high dwell time (Fig. 5D), still demonstrated no statistically significant difference. Additionally, equivalent results were observed when substituting the dwell time by the tRNA abundance (Fig. 5E). Again, a similar scenario was observed when we used dwell time and tRNA abundance obtained from hiPSC neuronal cell type (Fig. S6). We repeated this analysis now using the top 1000 mRNAs that were enriched in the CNOT3 precipitation (Fig. S7). Interesting, we observed a slight but statistically significant association between A‐site dwell time and mRNA stabilization after CNOT3 KO (Fig. S7A). However, this effect was not observed when we substituted the dwell time by the tRNA abundance (Fig. S7B,D). Moreover, when we used dwell time obtained from hiPSC neuronal cell type no difference was observed (Fig. S7C), resembling the results observed in Fig. 5. In summary, features such as decoding time (Fig. 5A) and tRNA abundance (Fig. 5E) of codons following PTMD codons do not appear to influence the half‐life of endogenous mRNAs after knockout of CNOT3.

### The position of PTMD codons on transcript do not explain the CNOT3 recruitment

We have shown that additional factors are likely involved in PTMD regulation; however, their nature remains unclear. We tested whether PTMD codon positions differ between genes that recruit CNOT3 and those that do not recruit it. No statistically significant difference was observed between the two groups (Fig. S8). Nevertheless, the distribution of PTMD codon positions deviated significantly from uniformity (Kolmogorov–Smirnov test, *P* = 8.86 × 10^−10^), likely reflecting the higher frequency of PTMD codons toward the 5′ end of transcripts (Fig. S8).

### PTMD‐rich and slow‐decoding transcripts are not the most affected under CNOT3 knockout

**Fig. 6:**
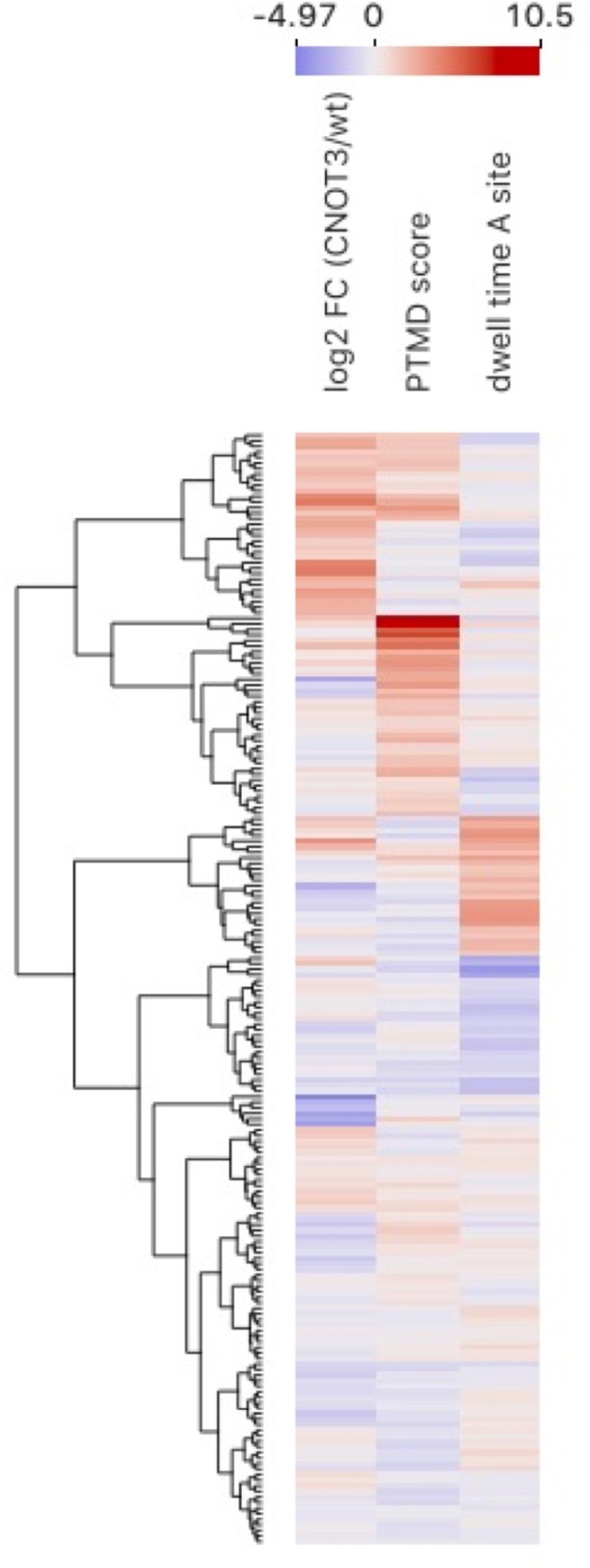
Cluster Analysis of PTMD score, mRNA half‐life change after CNOT3 knockout, and A‐site tAI or dwell time. Each 7639 transcripts was grouped in one of 200 groups (k means), according to their similarity on the three metrics. Each line represents the average values of the transcripts in the group. The number of transcripts in each group may vary. The values were normalized using Z‐scores for each variable.

After discussing how codon dwell time and tRNA abundance, in association with PTMD codons, affect a transcript's half‐life under CNOT3 knockout, we asked whether the transcripts most affected by CNOT3 knockout were the same we studied in the previous analysis. We then performed a cluster analysis comparing PTMD score, dwell time in the A‐site after a PTMD codon, and half‐life fold change after CNOT3 knockout, between 7639 transcripts, grouping them by how similar they were regarding these variables. For simplicity, 7639 transcripts were merged by k‐means and clustered into 200 groups with similar values for these variables (Fig. 6). Interestingly, the codons with the most half‐life change under CNOT3 knockout were clustered separately from those with the highest PTMD score and codon dwell time (Fig. 6), indicating that they don't occur concomitantly, and other features than the PTMD machinery must be responsible for most of the effects of CNOT3 on mRNA half‐life. This observation is consistent with the established role of CCR4‐NOT as a broad regulator of mRNA stability [36, 37, 38, 39, 40, 41, 42].

## Discussion

mRNA half‐life correlation to codon optimality is conserved across various eukaryotic species, from yeast to humans, although being less prominent in the last one. The CCR4‐NOT complex plays a key role in this correlation. Nevertheless, the complex seems to operate differently in humans than it does in yeast.

In yeast, the Not5 component of the CCR4‐NOT complex targets slow decoding codons in the A‐site. Codons with low tRNA availability are translated more slowly due to delayed codon‐anticodon pairing, which promotes a ribosomal conformation with both the E‐site and A‐site vacant—this conformation is required for Not5 binding. Upon binding, Not5 recruits the mRNA decay machinery, resulting in shorter half‐lives for transcripts enriched in slow‐decoding codons.

Conversely, in humans, CNOT3—the homolog of Not5—preferentially targets specific codons in the P‐site, particularly the AGG, CGA, and CGG codons of arginine. Although codon optimality is a major determinant of mRNA stability in yeast, its contribution to mRNA half‐life in humans is less well‐understood. Bazzini *et al*. [7] demonstrated that codon identity is a major factor in human mRNA stability, independently of the poly‐A tail. In contrast, Coller *et al*. highlighted the role of amino acid identity, in addition to codon bias, in mRNA regulation [26]. Alternatively, Agarwal and Kelley showed that other features contribute to the mRNA half‐life as much as codon bias, such as ORF exon junction density and 3′ UTR length [43].

We observed that, despite the transcripts richer in AGG, CGA, and CGG being more stable, this effect is highly independent from the CNOT3 effects through PTMD machinery, as proposed by Mendell [27]. Transcripts enriched in codon pairs where AGG, CGA, or CGG are immediately followed by a slow‐decoding codon do not exhibit altered stability upon CNOT3 knockout when compared to transcripts with similar AGG, CGA, and CGG densities but followed by a rapid‐decoding codon (Fig. 3A).

One possible explanation for this apparent discrepancy to Mendell's results is the amount of PTMD codons required to recruit the CCR4‐NOT complex. The minimal number of PTMD codons per transcript necessary to trigger a substantial mRNA degradation remains unknown. Mendell's group used a reporter containing 42 tripeptides centered on a PTMD arginine codon to demonstrate accelerated CNOT3‐mediated mRNA decay [27]. Interestingly, only 20 human genes closely resemble this reporter, containing at least 14 PTMD codons within a 42‐codon window (data not shown). The extent to which PTMD contributes to global mRNA half‐life regulation and the evolutionary basis for its specificity toward a subset of arginine codons remain to be elucidated.

## Conflict of interest

The authors declare no conflicts of interest.

## Author contributions

RLC and FLP conceived and designed the project. RLC obtained and analyzed the data. RLC and FLP interpreted the data and wrote the paper.

## Supporting information


**Fig. S1.** Half‐life fold change under CNOT3 knockout over the wild‐type half‐life, in log2. The two groups were stratified the same way as Fig. 1, but excluding any transcript addressed as mitochondrial by its gene ontology. *P*‐value given by Kolmogorov–Smirnov's test. The top whisker, top of the box, line inside the box, bottom of the box, and bottom whisker represent the maximum value, the upper quartile (75th percentile), the median, the lower quartile (25th percentile), and the minimum value, respectively.
**Fig. S2**. Codon enrichment values for every codon in the A, P, and E sites, of ribo‐seq of ribosomes under CNOT3 immunoprecipitation, compared to standard ribo‐seq. As expected, the codons with highest enrichment scores in the P site were CGG, CGA and AGG (red points). This pattern didn't follow in the E and A sites. The enrichment scores of those codons were also higher in the P‐site than in E and A sites, even when compared to the most enriched codons in each site.
**Fig. S3**. Genes enriched under CNOT3 immunoprecipitation. Volcano plot of the enrichment of genes. Each point is a gene. X‐axis represents the log2 of the fold change between the ribosome profiling data under CNOT3 immunoprecipitation, over the standard ribosome profiling data. The genes to the right of the second line are the ones that were 2 times more abundant under CNOT3‐IP. The Y‐axis represent the adjusted *P*‐value for that gene, by Fisher's exact test.
**Fig. S4**. Pearson's correlation coefficient between codon pair enrichment and tRNA abundance of the first codon of the pair (A) when the second codon of the pair is a PTMD codon, or (B) when the second codon of the pair is not a PTMD codon. P‐values given by Pearson's correlation index (r).
**Fig. S5**. Correlations of PTMD occurrence, ribosome dwell time, and tRNA. (A) Correlation between codon pair occurrence in the genome ORFs, when the first codon of the pair is a PTMD codon, and the dwell time of the second codon in the pair. (B) Correlation between codon pair occurrence in the genome ORFs, when the first codon of the pair is a codon other than a PTMD one, and the dwell time of the second codon in the pair. (C) Correlation between codon pair enrichment in the genome ORFs, when the first codon of the pair is a PTMD codon, and the tRNA count for the anticodon correspondent to the second codon in the pair. (D) Correlation between codon pair occurrence in the genome ORFs, when the first codon of the pair is a codon other than a PTMD one, and the tRNA abundance for the anticodon correspondent to the second codon in the pair. Dwell time and tRNA abundance were obtained from [33]. *P*‐values given by Pearson's correlation index (r).
**Fig. S6**. Dwell time and tRNA availability of codons on the A‐site have no impact on P‐site tRNA‐mediated decay. (A) mRNA half‐life fold change (ΔCNOT3/WT) of transcripts with high PTMD score comparing the lowest dwell time in the A‐site after CGA, CGG, or AGG vs transcripts with the highest dwell time in the A‐site after CGA, CGG, or AGG. (B) mRNA half‐life fold change (ΔCNOT3/WT) of transcripts with high PTMD score, comparing the median dwell time for all codons throughout the transcript, instead of only the codons on the A‐site following PTMD codons. (C) mRNA half‐life fold change (ΔCNOT3/WT) of transcripts with high PTMD score, comparing transcripts with low dwell time on the A‐sites following PTMD codons to the transcripts with low median dwell time for all the codons throughout the transcript. (D) mRNA half‐life fold change (ΔCNOT3/WT) of transcripts with high PTMD score, comparing transcripts with high dwell time on the A‐sites following PTMD codons to the transcripts with high median dwell time for all the codons throughout the transcript. (E) Same analysis as (A) but using tRNA abundance in place of dwell time. *P*‐values given by Kolmogorov–Smirnov test. Dwell time and tRNA abundance were obtained from [33]. The top whisker, top of the box, line inside the box, bottom of the box, and bottom whisker represent the maximum value, the upper quartile (75th percentile), the median, the lower quartile (25th percentile), and the minimum value, respectively.
**Fig. S7**. Dwell Time and tRNA availability of codons on the A‐site have no impact on P‐site tRNA‐mediated decay. (A) mRNA half‐life fold change (ΔCNOT3/WT) of the 1000 top CNOT3 IP group of genes with all the genes (4481) considered as not enriched under CNOT3‐IP comparing the lowest dwell time in the A‐site after CGA, CGG or AGG vs transcripts with the highest dwell time in the A‐site after CGA, CGG or AGG. (B) Same analysis as (A) but using tRNA abundance in place of dwell time. *P*‐values given by Kolmogorov–Smirnov test. Dwell time and tRNA abundance of A and B were obtained from [31, 32] respectively. Dwell time and tRNA abundance of C and D were obtained from [33]. The top whisker, top of the box, line inside the box, bottom of the box, and bottom whisker represent the maximum value, the upper quartile (75th percentile), the median, the lower quartile (25th percentile), and the minimum value, respectively.
**Fig. S8**. Relative PTMD positions in the gene. X‐axis represents the gene relative position, being 0.0 the initiation ATG, and 1.0 the stop codon. The Y‐axis represents the frequency of each PTMD codon found in the gene. The numbers in the box on the right represent the PTMD codons found in CNOT3 IP group vs the control. The Mann–Whitney compare the PTMD frequency on gene relative position between PTMD codons found in CNOT3 IP group vs the control.

**Table S1.** Table containing data on codon pair‐level resolution, used for Figures 4, S4 and S5.

**Table S2.** Table containing data on the gene‐level resolution, used for Figures 1, 2, 3, 5, 6, S1, S3, S6, S7

## Data Availability

All data generated or analyzed during this study are included in this published article and its Supporting Information files. The algorithms used during the current study are available from the corresponding author upon reasonable request.
