# Supplementary material for: Transcripts enriched in codons that trigger P‐site tRNA‐mediated mRNA decay possess stable mRNA
Source: FEBS Open Bio. 2026 Jun 12:10.1002/2211-5463.70277. Online ahead of print. doi: 10.1002/2211-5463.70277 (PMC13398649; doi:10.1002/2211-5463.70277)
Supplement: Supplementary file 1 — Fig. S1. Half‐life fold change under CNOT3 knockout over the wild‐type half‐life, in log2. The two groups were stratified the same way as Fig. 1, but excluding any transcript addressed as mitochondrial by its gene ontology. P‐value given by Kolmogorov–Smirnov's test. The top whisker, top of the box, line inside the box, bottom of the box, and bottom whisker represent the maximum value, the upper quartile (75th percentile), the median, the lower quartile (25th percentile), and the minimum value, respectively. Fig. S2. Codon enrichment values for every codon in the A, P, and E sites, of ribo‐seq of ribosomes under CNOT3 immunoprecipitation, compared to standard ribo‐seq. As expected, the codons with highest enrichment scores in the P site were CGG, CGA and AGG (red points). This pattern didn't follow in the E and A sites. The enrichment scores of those codons were also higher in the P‐site than in E and A sites, even when compared to the most enriched codons in each site. Fig. S3. Genes enriched under CNOT3 immunoprecipitation. Volcano plot of the enrichment of genes. Each point is a gene. X‐axis represents the log2 of the fold change between the ribosome profiling data under CNOT3 immunoprecipitation, over the standard ribosome profiling data. The genes to the right of the second line are the ones that were 2 times more abundant under CNOT3‐IP. The Y‐axis represent the adjusted P‐value for that gene, by Fisher's exact test. Fig. S4. Pearson's correlation coefficient between codon pair enrichment and tRNA abundance of the first codon of the pair (A) when the second codon of the pair is a PTMD codon, or (B) when the second codon of the pair is not a PTMD codon. P‐values given by Pearson's correlation index (r). Fig. S5. Correlations of PTMD occurrence, ribosome dwell time, and tRNA. (A) Correlation between codon pair occurrence in the genome ORFs, when the first codon of the pair is a PTMD codon, and the dwell time of the second codon in the pair. (B) Correlation betwe [file FEB4-9999-0-s001.docx]

**Transcripts enriched in codons that trigger P-site tRNA-mediated mRNA decay possess stable mRNA**

Rodolfo L. Carneiro and Fernando L. Palhano*

Programa de Biologia Estrutural, Instituto de Bioquímica Médica Leopoldo de Meis, Universidade Federal do Rio de Janeiro, Rio de Janeiro, RJ, 21941-902, Brazil.

* To whom correspondence should be addressed: Tel.: +55 21 3938-6761; Email: [palhano@bioqmed.ufrj.br](mailto:palhano@bioqmed.ufrj.br)


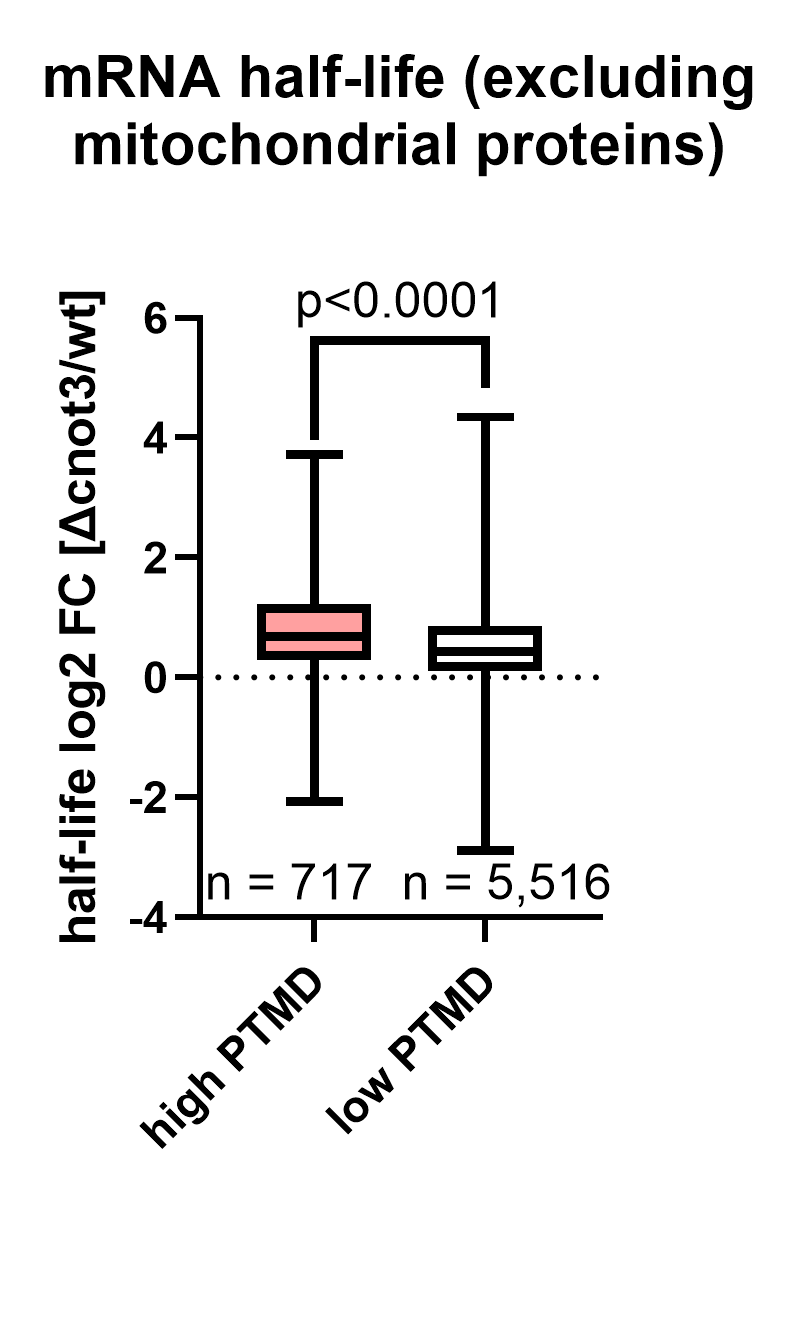


**Figure S1. Half-life fold change under CNOT3 knockout over the wild-type half-life, in log2.** The two groups were stratified the same way as figure 1, but excluding any transcript addressed as mitochondrial by its gene ontology. p-value given by Kolmogorov-Smirnov’s test. The top whisker, top of the box, line inside the box, bottom of the box, and bottom whisker represent the maximum value, the upper quartile (75th percentile), the median, the lower quartile (25th percentile), and the minimum value, respectively.


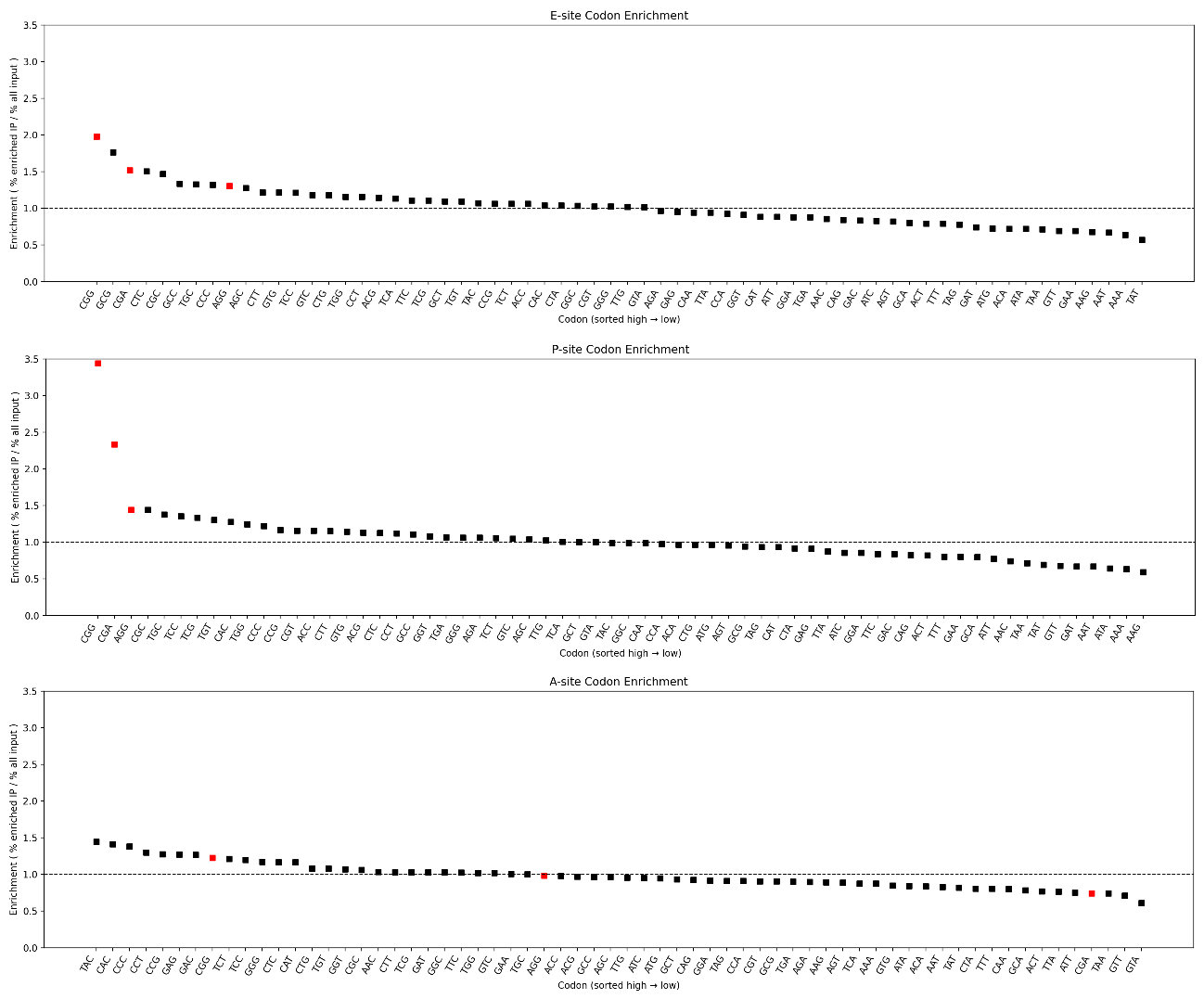


**Figure S2. Codon enrichment values for every codon in the A, P, and E sites, of ribo-seq of ribosomes under CNOT3 immunoprecipitation, compared to standard ribo-seq.** As expected, the codons with highest enrichment scores in the P site were CGG, CGA and AGG (red points). This pattern didn’t follow in the E and A sites. The enrichment scores of those codons were also higher in the P-site than in E and A sites, even when compared to the most enriched codons in each site.


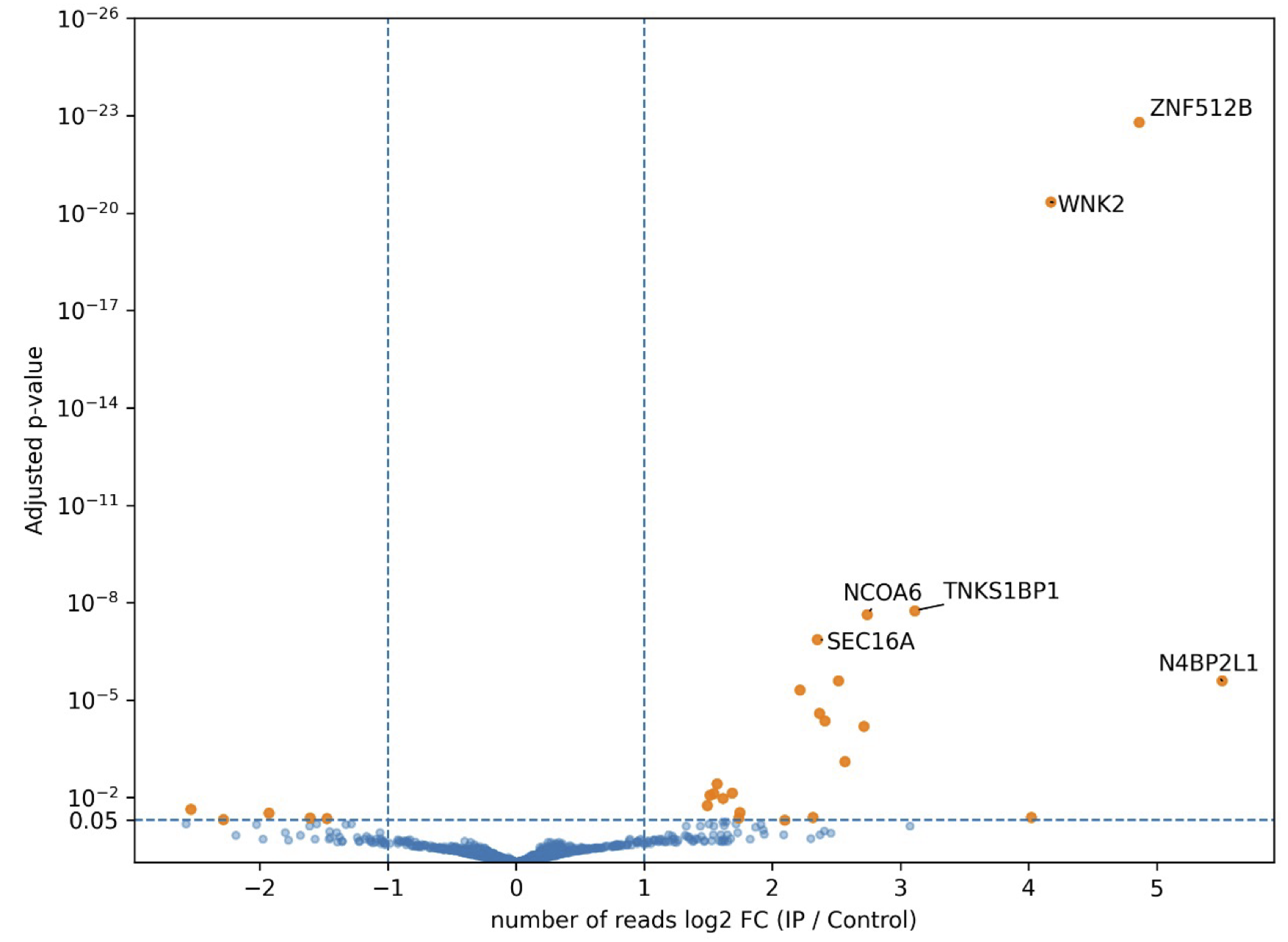


**Figure S3. Genes enriched under CNOT3 immunoprecipitation.** Volcano plot of the enrichment of genes. Each point is a gene. X-axis represents the log2 of the fold change between the ribosome profiling data under CNOT3 immunoprecipitation, over the standard ribosome profiling data. The genes to the right of the second line are the ones that were 2 times more abundant under CNOT3-IP. The Y-axis represent the adjusted p-value for that gene, by Fisher’s exact test.

**Figure S4. Pearson’s correlation coefficient between codon pair enrichment and tRNA abundance of the first codon of the pair (A)** when the second codon of the pair is a PTMD codon, or **(B)** when the second codon of the pair is not a PTMD codon. p-values given by Pearson’s correlation index (r).

**
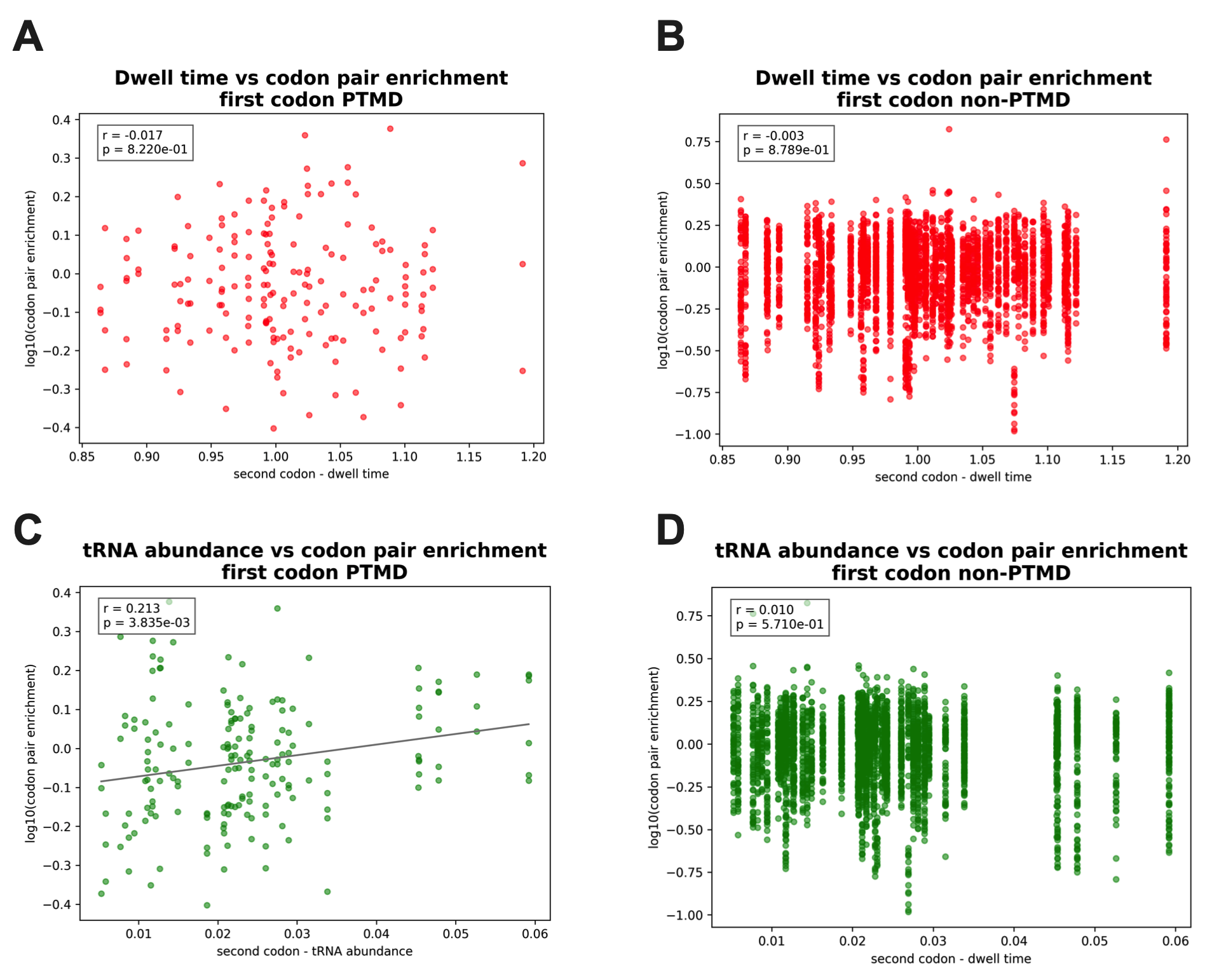
**

**Figure S5. Correlations of PTMD occurrence, ribosome dwell time, and tRNA. (A)** Correlation between codon pair occurrence in the genome ORFs, when the first codon of the pair is a PTMD codon, and the dwell time of the second codon in the pair. **(B)** Correlation between codon pair occurrence in the genome ORFs, when the first codon of the pair is a codon other than a PTMD one, and the dwell time of the second codon in the pair. **(C)** Correlation between codon pair enrichment in the genome ORFs, when the first codon of the pair is a PTMD codon, and the tRNA count for the anticodon correspondent to the second codon in the pair. **(D)** Correlation between codon pair occurrence in the genome ORFs, when the first codon of the pair is a codon other than a PTMD one, and the tRNA abundance for the anticodon correspondent to the second codon in the pair. Dwell time and tRNA abundance were obtained from [33]. p-values given by Pearson’s correlation index (r).

**
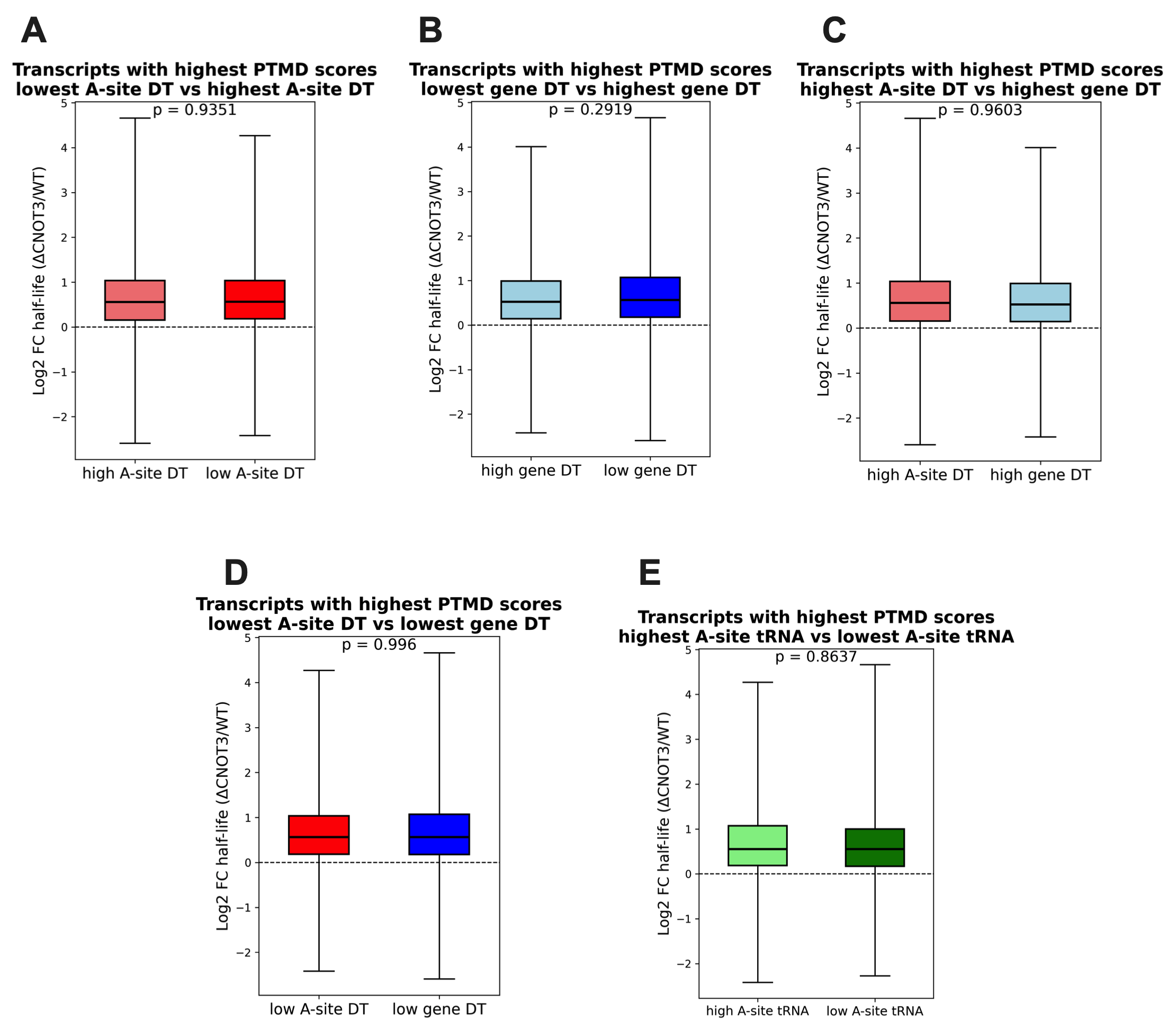
**

**Figure S6. Dwell Time and tRNA availability of codons on the A-site have no impact on P-site tRNA Mediated Decay.** (A) mRNA half-life fold change (ΔCNOT3/WT) of transcripts with high PTMD score comparing the lowest dwell time in the A-site after CGA, CGG or AGG vs transcripts with the highest dwell time in the A-site after CGA, CGG or AGG. (B) mRNA half-life fold change (ΔCNOT3/WT) of transcripts with high PTMD score, comparing the median dwell time for all codons throughout the transcript, instead of only the codons on the A-site following PTMD codons. (C) mRNA half-life fold change (ΔCNOT3/WT) of transcripts with high PTMD score, comparing transcripts with low dwell time on the A-sites following PTMD codons to the transcripts with low median dwell time for all the codons throughout the transcript. (D) mRNA half-life fold change (ΔCNOT3/WT) of transcripts with high PTMD score, comparing transcripts with high dwell time on the A-sites following PTMD codons to the transcripts with high median dwell time for all the codons throughout the transcript. (E) Same analysis as (A) but using tRNA abundance in place of dwell time. p-values given by Kolmogorov-Smirnov test. Dwell time and tRNA abundance were obtained from [33]. The top whisker, top of the box, line inside the box, bottom of the box, and bottom whisker represent the maximum value, the upper quartile (75th percentile), the median, the lower quartile (25th percentile), and the minimum value, respectively.


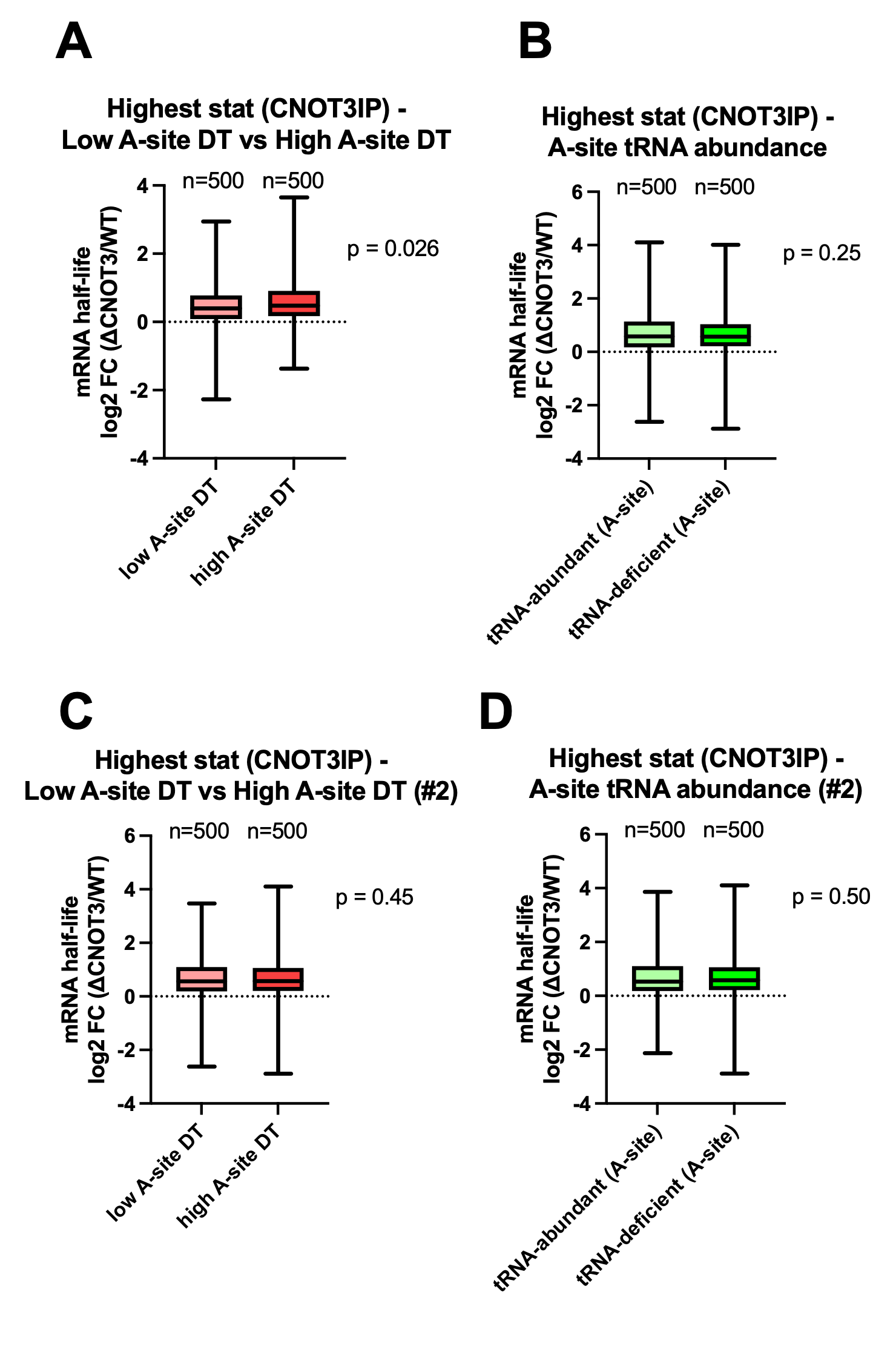


**Figure S7. Dwell Time and tRNA availability of codons on the A-site have no impact on P-site tRNA Mediated Decay.** (A) mRNA half-life fold change (ΔCNOT3/WT) of the 1,000 top CNOT3 IP group of genes with all the genes (4,481) considered as not enriched under CNOT3-IP comparing the lowest dwell time in the A-site after CGA, CGG or AGG vs transcripts with the highest dwell time in the A-site after CGA, CGG or AGG. (B) Same analysis as (A) but using tRNA abundance in place of dwell time. p-values given by Kolmogorov-Smirnov test. Dwell time and tRNA abundance of A and B were obtained from [31] and [32] respectively. Dwell time and tRNA abundance of C and D were obtained from [33]. The top whisker, top of the box, line inside the box, bottom of the box, and bottom whisker represent the maximum value, the upper quartile (75th percentile), the median, the lower quartile (25th percentile), and the minimum value, respectively.


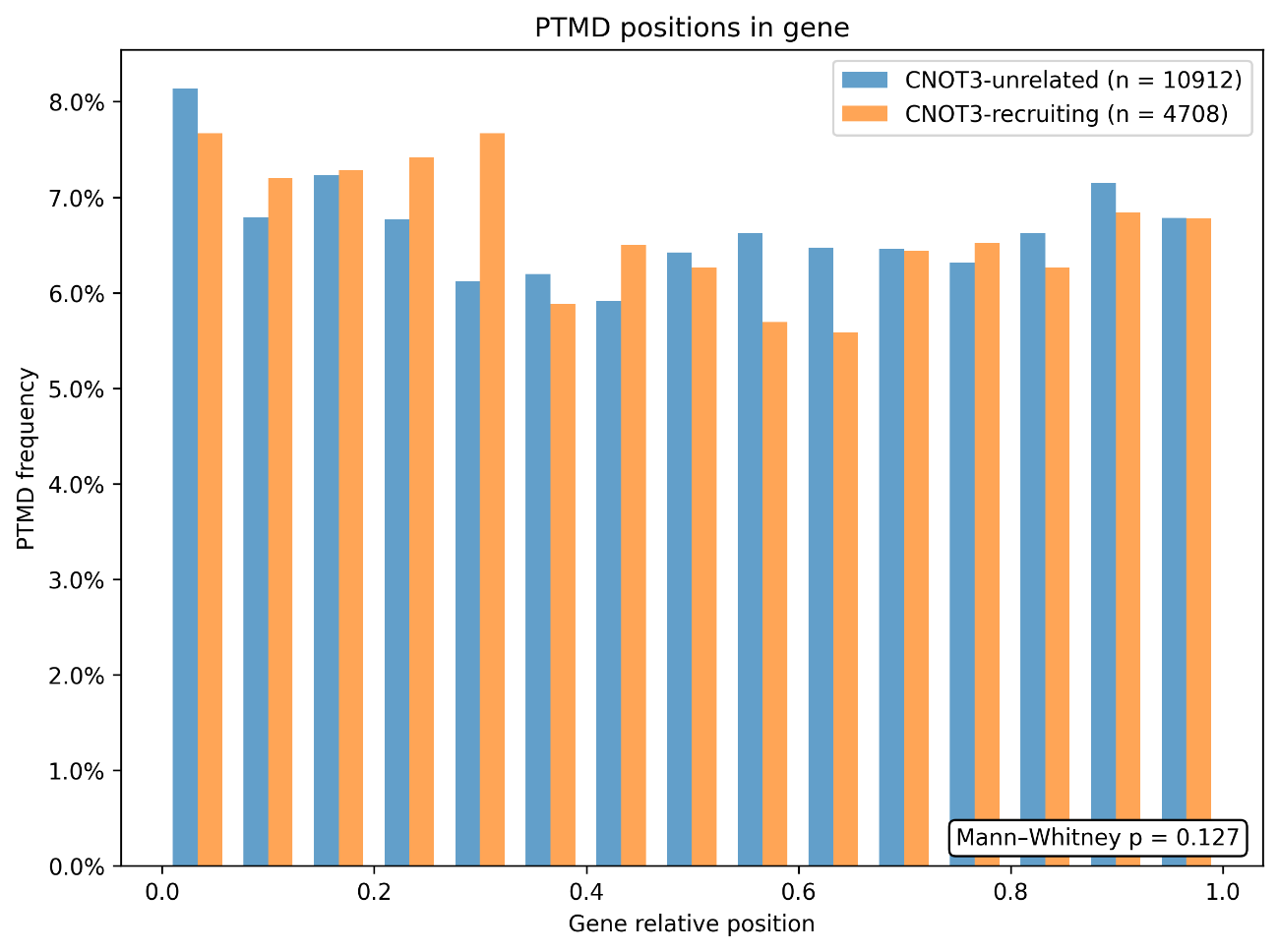


**Figure S8. Relative PTMD positions in the gene.** X-axis represents the gene relative position, being 0.0 the initiation ATG, and 1.0 the stop codon. The Y-axis represents the frequency of each PTMD codon found in the gene. The numbers in the box on the right represent the PTMD codons found in CNOT3 IP group vs the control. The Mann-Whitney compare the PTMD frequency on gene relative position between PTMD codons found in CNOT3 IP group vs the control.
